# Residual Inflammatory Risk and its Association With Events in East Asian Patients After Coronary Intervention

**DOI:** 10.1016/j.jacasi.2021.11.014

**Published:** 2022-04-12

**Authors:** Jong-Hwa Ahn, Udaya S. Tantry, Min Gyu Kang, Hyun Woong Park, Jin-Sin Koh, Jae Seok Bae, Sang Young Cho, Kye-Hwan Kim, Jeong Yoon Jang, Jeong Rang Park, Yongwhi Park, Seok-Jae Hwang, Choong Hwan Kwak, Jin-Yong Hwang, Paul A. Gurbel, Young-Hoon Jeong

**Affiliations:** a Department of Internal Medicine, Gyeongsang National University School of Medicine and Cardiovascular Center, Gyeongsang National University Changwon Hospital, Changwon, South Korea; b Sinai Center for Thrombosis Research and Drug Development, Sinai Hospital of Baltimore, Baltimore, Maryland, USA; c Department of Internal Medicine, Gyeongsang National University School of Medicine and Gyeongsang National University Hospital, Jinju, South Korea; d Institute of the Health Sciences, Gyeongsang National University, Jinju, South Korea

**Keywords:** coronary artery disease, C-reactive protein, East Asian, residual inflammation, AMI, acute myocardial infarction, ASCVD, atherosclerotic cardiovascular disease, CAD, coronary artery disease, CKD, chronic kidney disease, hsCRP, high sensitivity C-reactive protein, LDL-C, low-density lipoprotein cholesterol, MACE, major adverse cardiovascular events, PCI, percutaneous coronary intervention, RIR, residual inflammatory risk

## Abstract

**Background:**

East Asian population has a low level of inflammation compared with Western population. The prognostic implication of residual inflammatory risk (RIR) remains uncertain in East Asians.

**Objectives:**

This study sought to provide an analysis to estimate early-determined RIR and its association with clinical outcomes in East Asian patients with coronary artery disease (CAD).

**Methods:**

In an East Asian registry including patients with CAD undergoing percutaneous coronary intervention (PCI) (n = 4,562), RIR status was determined by measuring high-sensitivity C-reactive protein (hsCRP) serially at admission and at 1-month follow-up. Patients were stratified into 4 groups according to hsCRP criteria (≥2 mg/L): 1) persistent low RIR (low_on admission_-low_1 month_: 51.0%); 2) fortified RIR (low_on admission_-high _1 month_: 10.3%); 3) attenuated RIR (high_on admission_-low_1 month_: 20.5%); and 4) persistent high RIR (high_on admission_-high_1 month_: 18.3%). The risks of all-cause death, ischemic events, and major bleeding were evaluated.

**Results:**

In our cohort, median levels of hsCRP were significantly decreased over time (1.3 to 0.9 mg/L; *P* < 0.001). Compared with hsCRP on admission, hsCRP at 1 month showed the greater associations with all-cause death and ischemic event. During clinical follow-up, risks of clinical events were significantly different across the groups (log-rank test, *P* < 0.001). Compared with other RIR groups, persistent high RIR showed the higher risk for all-cause death (HR_adjusted_, 1.92; 95% CI: 1.44 to 2.55; *P* < 0.001), ischemic events (HR_adjusted_, 1.26; 95% CI: 1.02 to 1.56; *P* = 0.032), and major bleeding (HR_adjusted_, 1.98; 95% CI: 1.30 to 2.99; *P* < 0.001), respectively.

**Conclusions:**

Approximately one-fifth of East Asian patients with CAD have persistent high RIR, which shows the close association with occurrence of ischemic and bleeding events. (Gyeongsang National University Hospital Registry [GNUH]; NCT04650529)

Systemic and vascular inflammation plays crucial biological roles in the progression and destabilization of atherosclerosis, occurrence of atherothrombotic events,^1^ and long-term clinical outcomes.^2^^,^^3^ Numerous clinical and experimental evidences have supported usefulness of high-sensitivity C-reactive protein (hsCRP) in assessing inflammatory level and predicting clinical outcomes in healthy individuals^4^ or patients with cardiovascular disease (CVD).^2^^,^^5^^,^^6^

Despite contemporary evidence-based lifestyle interventions and pharmacologic strategies achieving a targeted level of low-density lipoprotein cholesterol (LDL-C), clinical events derived from atherosclerotic cardiovascular disease (ASCVD) are substantially maintained.^7^^,^^8^ A biological pathway associated with residual CV risk, in many patients with CVD having the recommended LDL-C levels, has focused on controlling residual systemic inflammation. In addition, the phenotype of residual inflammatory risk (RIR) has been become more important than before, as therapeutic strategies to control inflammation are emerging,^9^ and several interventions could reduce the risk of CV events.^10^, ^11^, ^12^

It is very important to determine the reliable strategy to select patients with CVD with realistic RIR and introduction of the right therapy for these patients at the right time.^13^ The levels of hsCRP can be dynamically changed over the early phase in unstable patients. Therefore, different measuring timing may explain inconsistencies of its clinical implication in clinical data. Serial assessment of inflammation status has been suggested to decide the reliable RIR phenotype in patients with coronary artery disease (CAD),^14^, ^15^, ^16^ but clinical usefulness in early determination of RIR following percutaneous coronary intervention (PCI) remains uncertain.

Compared with Western patients, East Asian patients have shown a lower risk of post-PCI atherothrombotic complications.^17^^,^^18^ A low level of thrombogenicity in East Asian vs other races may be a crucial factor to account for this “East Asian Paradox.”^18^ It is intriguing that there are significant racial differences in inflammatory activity (eg, African Americans > East Asians), but its clinical implication according to the race has been remained uncertain.^19^ Therefore, we performed the cohort analysis to validate early determination of RIR phenotype and its association with long-term clinical outcomes after PCI in East Asian patients with CAD.

## Methods

### Study design

The study population was derived from the G-NUH (Gyeongsang-National University Hospitals; NCT04650529) registry, which was a prospective 2-center database that enrolled PCI-treated patients with significant CAD (Jinju and Changwon) and evaluated multiple hemostatic, vascular, and physiologic parameters if indicated.^20^ In this retrospective analysis, we enrolled PCI-treated patients with available on-admission hs-CRP measurement between January 2010 and November 2018 (n = 5,840).

**Figure 1:**
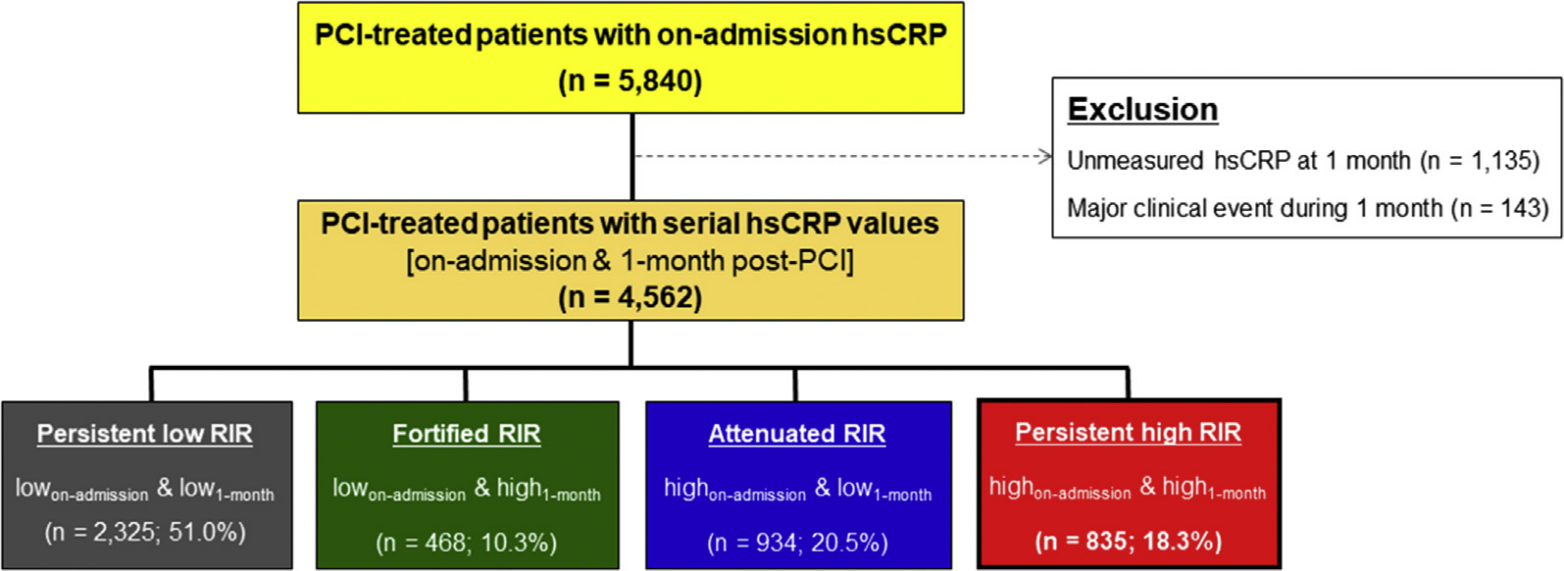
Study Flow Diagram hsCRP = high-sensitivity C-reactive protein; PCI = percutaneous coronary intervention; RIR = residual inflammatory risk.

Patients were eligible for this analysis if they had serial hs-CRP measurements (at admission and 1-month follow-up post-PCI) and did not experience major ischemic or bleeding events during 1 month after the PCI procedure (Figure 1). Baseline demographic, angiographic, and procedural characteristics and clinical outcome data were collected prospectively. Patients were routinely followed at 6 and 12 months after the PCI procedure and annually thereafter. Further information was collected through medical records or by telephone contact, if necessary. The institutional review board of the hospital approved the registry and waived the requirement for written informed consent for access to an institutional registry. The study was performed in accordance with the Good Clinical Practice Guidelines and the principles of the Declaration of Helsinki.

### hsCRP measurement and population group

hsCRP was measured twice (on admission and 1-month follow-up after PCI). Baseline biochemical assessment, including hsCRP, was performed in whole blood samples drawn immediately after arrival to the emergency department or general ward. The hsCRP level was measured with a commercially available enzyme-linked immunosorbent assay using UniCel DxC 800 Synchron Clinical System (Beckman Coulter, Inc.). Other biochemical measurements, including complete blood count, lipid profile, and chemistry profile, were performed using standard laboratory assays. At 1-month (± 5 days) visit, follow-up hsCRP measurement was performed using blood collected from the antecubital vein at 2 to 6 hours after the last drug administration.

Patients were stratified into 4 groups according to hsCRP cutoff of 2 mg/L:^10^^,^^14^ 1) persistent low RIR (low_on admission_ and low_1 month_); 2) fortified RIR (low_on admission_ and high_1 month_); 3) attenuated RIR (high_on admission_ and low_1 month_); and 4) persistent high RIR (high_on admission_ and high_1 month_).

### Clinical outcomes

The primary endpoint of this analysis was occurrence of all-cause death after 1-month post-PCI. Secondary endpoints were major adverse cardiovascular events (MACE), CV death, myocardial infarction (MI), or cerebrovascular accident (CVA) and major bleeding after 1-month post-PCI. We also evaluated the main determinants of persistent high RIR and its clinical impact according to the underlying risk.

All deaths were considered to be of CV cause unless a definite non-CV cause could be established. Acute MI (AMI) was defined as increased cardiac troponin values with ischemic symptoms or ischemic changes on electrocardiogram or imaging evidence of recent loss of viable myocardium or new regional wall-motion abnormality that were not related to procedure.^21^ CVA was defined as evidence of neurologic deficit requiring hospitalization and with clinically documented lesions on brain computed tomography or magnetic resonance imaging. In addition, major bleeding was defined as Bleeding Academic Research Consortium (BARC) bleeding type 3 or 5.^22^

### Statistical analysis

The Kolmogorov-Smirnov test was performed to analyze the normal distribution of continuous variables. Continuous variables were expressed as mean ± SD or as median (IQR), whereas categorical variables were presented as absolute numbers and frequencies (%). The Student's unpaired *t*-test for parametric continuous variables and the Mann-Whitney U test for nonparametric continuous variables were used. Comparisons between categorical variables were performed using the Pearson chi-square test or Fisher exact test when the Cochran rule was not met for categorical variables.

All demographic characteristics and laboratory measurements were evaluated in a univariate analysis for predicting presence of persistent high RIR. Variables with *P* < 0.10 in univariable analysis were then entered into multivariable logistic regression with backward elimination providing OR and 95% CI. Univariable and multivariable Cox proportional hazard analysis were performed to estimate hazard ratios for all-cause death/MACE/major bleeding among RIR types and to adjust for known potential confounders (index MI presentation, age, gender, body mass index [BMI], smoking, diabetes mellitus [DM], hypertension, cholesterol level, chronic kidney disease [CKD], hemoglobin, previous stroke, left ventricular ejection fraction [LVEF], PCI for left anterior descending [LAD] artery lesion, multivessel disease, use of drug-eluting stents [DES], potent P2Y_12_ inhibitor, beta blocker, angiotensin blockade, and statin). A *P* value <0.05 was considered statistically significant. All statistical analyses were done with IBM/SPSS version 24.0 (IBM SPSS Statistics).

## Results

Of the 5,840 patients from the initial cohort, those with clinical event during 1-month follow-up (n = 143) and subjects without 1-month hsCRP measurement (n = 1,135) were excluded, including 4,562 patients (78.1%) with serial hsCRP measurements in the final analysis (Figure 1). Mean age in the final cohort was 65.3 ± 11.7 years. Approximately one-half of the patients were initially presented with AMI (57.1%) and mostly treated with drug-eluting stents (89.5%).

**Figure 2:**
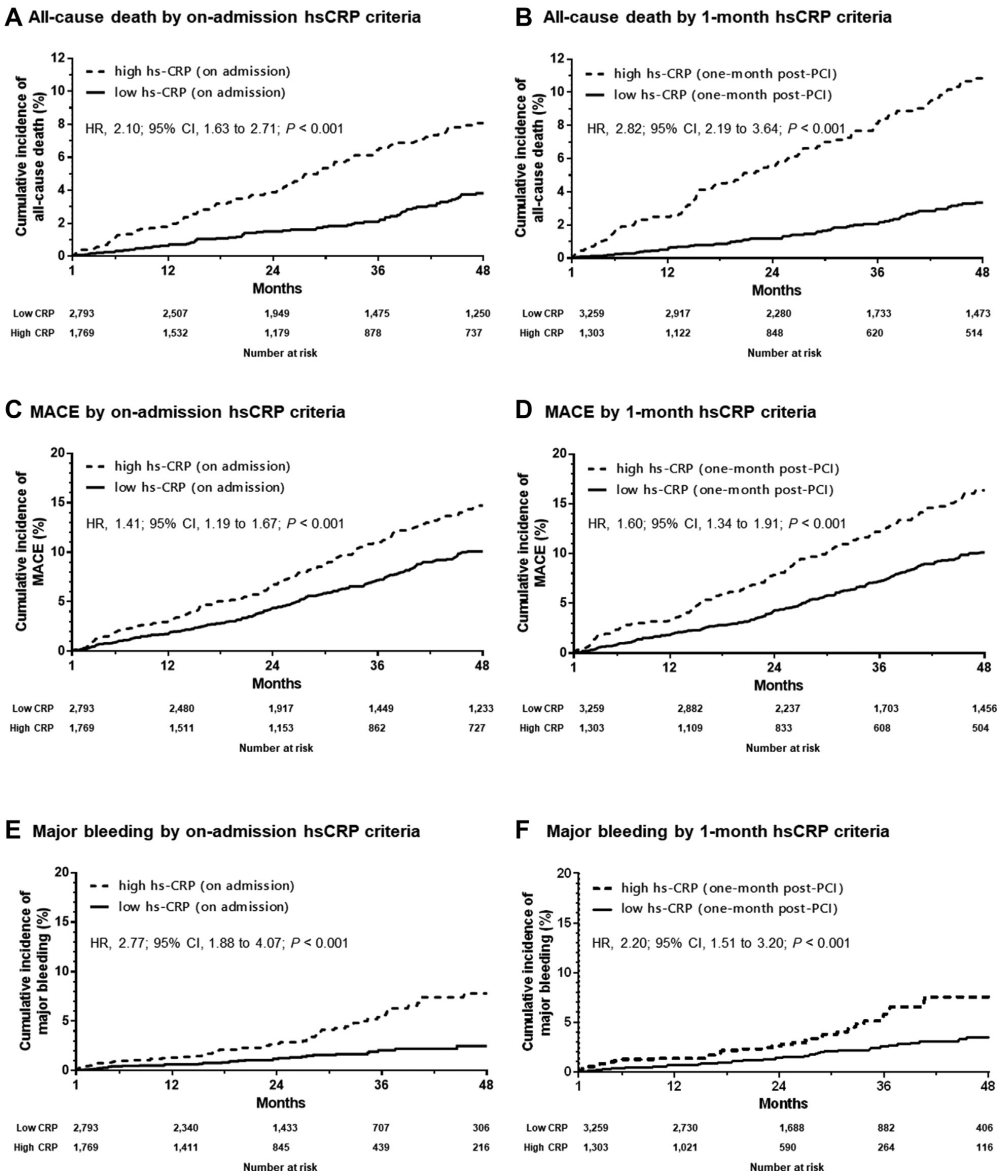
Kaplan-Meier Curves for Adverse Clinical Events, According to hsCRP Criteria at on Admission and at 1 Month Major adverse cardiovascular event (MACE) included cardiovascular (CV) death, myocardial infarction (MI), and cerebrovascular accident (CVA). Abbreviations as in Figure 1.

The hsCRP levels were significantly decreased from 1.3 (IQR: 0.5 to 3.8) mg/L at admission to 0.9 (IQR: 0.5-2.3) mg/L at 1-month follow-up (*P* < 0.001). During a median follow-up duration of 36.0 (IQR: 18.9 to 71.9) months, a total of 238 cases of all-cause death (5.2%), 522 MACEs (11.4%) (92 CV deaths [2.0%], 272 nonfatal MIs [6.0%], 158 nonfatal CVAs [3.5%]), and 111 cases of major bleeding (2.4%) occurred. Compared with the criteria of high hsCRP on admission (HR: 2.10; 95% CI: 1.63 to 2.71; *P* < 0.001) (Figure 2A), the criteria of high hsCRP at 1 month (HR: 2.82; 95% CI: 2.19 to 3.64; *P* < 0.001) (Figure 2B) was more predictive of all-cause death. This trend was similar in terms of association between high hsCRP criteria and occurrence of MACE (on admission: HR, 1.41; 95% CI: 1.19 to 1.67; *P* < 0.001 vs 1 month: HR: 1.60; 95% CI: 1.34 to 1.91; *P* < 0.001) (Figures 2C to 2D).

### Baseline characteristics according to type of residual inflammatory risk

According to serial hsCRP measurements, 2,325 patients were classified as persistent low RIR (51.0%), 468 fortified RIR (10.3%), 934 attenuated RIR (12.1%), and 835 persistent high RIR (18.3%) (Figure 1). Patents with persistent high RIR had higher prevalence of DM and CKD and were more frequently current smokers and presented with MI compared with the other groups (Table 1). In addition, those patients showed higher levels of white blood cell (WBC) count and total cholesterol level and had higher prevalence of anemia and multivessel disease. On multivariable logistic regression analysis, persistent high RIR phenotype was independently associated with age (per 1 year increase: OR: 1.02; 95% CI: 1.01 to 1.02; *P* < 0.001), smoking (OR: 1.57; 95% CI: 1.30 to 1.89; *P* < 0.001), CKD (OR: 1.91; 95% CI: 1.55 to 2.35; *P* < 0.001), hemoglobin (per 1 g/dL increase: OR: 0.90; 95% CI: 0.85 to 0.94; *P* < 0.001), WBC count (per 10^3^/mm^3^ increase: OR: 1.07; 95% CI: 1.04 to 1.09; *P* < 0.001), HDL-cholesterol (per 1 mg/dL increase: OR: 0.99; 95% CI: 0.98 to 0.99; *P* < 0.001), LDL-cholesterol (per 1 mg/dL increase: OR: 1.00; 95% CI: 1.00 to 1.01; *P* < 0.001), multivessel disease (OR: 1.30; 95% CI: 1.10 to 1.52; *P* = 0.002) and discharge medication of statin (OR: 0.65; 95% CI: 0.46-0.92; *P* < 0.001) (Table 2).

**Table 1:** Baseline Characteristics According to Type of Residual Inflammatory Risk

	Overall Population (N = 4,562)	Persistent Low RIR (n = 2,325)	Fortified RIR (n = 468)	Attenuated RIR (n = 934)	Persistent High RIR (n = 835)	*P* Value
On-admission hsCRP, mg/L	7.5 ± 25.3	0.7 ± 0.5	0.9 ± 0.5	16.1 ± 35.0	20.4 ± 41.4	<0.001
Median (IQR)	1.3 (0.5-3.8)	0.6 (0.4-1.0)	0.9 (0.5-1.3)	4.8 (2.9-11.4)	5.9 (3.3-16.7)	<0.001
1-month hsCRP, mg/L	3.9 ± 12.9	0.7 ± 0.4	9.3 ± 14.8	0.9 ± 0.5	13.2 ± 25.4	<0.001
Median (IQR)	0.9 (0.5-2.3)	0.6 (0.4-0.9)	3.9 (2.5-7.6)	0.8 (0.5-1.2)	4.9 (2.9-11.1)	<0.001
Index presentation						<0.001
Stable angina	1,531 (33.6)	845 (36.3)	173 (37.0)	280 (30.0)	233 (27.9)	
Unstable angina	427 (9.4)	251 (10.8)	39 (8.3)	71 (7.6)	66 (7.9)	
NSTEMI	1,414 (31.0)	642 (27.6)	136 (29.1)	332 (35.5)	304 (36.4)	
STEMI	1,190 (26.1)	587 (25.2)	120 (25.6)	251 (26.9)	232 (27.8)	
Age, y	65.3 ± 11.7	64.2 ± 11.3	66.8 ± 11.9	65.6 ± 11.7	67.2 ± 12.3	<0.001
Male	3,222 (70.6)	1643 (70.7)	335 (71.6)	647 (69.3)	597 (71.5)	0.720
Body mass index, kg/m^2^	24.3 ± 3.5	24.4 ± 3.3	23.8 ± 3.3	24.3 ± 3.5	24.1 ± 4.0	0.022
Risk factors						
Smoking	1,473 (32.3)	676 (29.1)	162 (34.6)	319 (34.2)	316 (37.8)	<0.001
Diabetes mellitus	1,405 (30.8)	690 (29.7)	137 (29.3)	295 (31.6)	283 (33.9)	0.116
Hypertension	2,387 (52.3)	1216 (52.3)	241 (51.5)	493 (52.8)	437 (52.3)	0.976
Dyslipidemia	2,444 (53.6)	1256 (54.0)	264 (56.4)	481 (51.5)	443 (53.1)	0.333
Chronic kidney disease	739 (16.2)	262 (11.3)	74 (15.8)	166 (17.8)	237 (28.4)	<0.001
Anemia	1,329 (29.1)	560 (24.1)	122 (26.1)	312 (33.4)	335 (40.1)	<0.001
Previous history						
Previous MI	274 (6.0)	157 (6.8)	34 (7.3)	54 (5.8)	29 (3.5)	0.004
Previous PCI	671 (14.7)	377 (16.2)	74 (15.8)	123 (13.2)	97 (11.6)	0.005
Previous CABG	26 (0.6)	16 (0.7)	1 (0.2)	7 (0.7)	2 (0.2)	0.288
Previous stroke	316 (6.9)	146 (6.3)	28 (6.0)	70 (7.5)	72 (8.6)	0.096
Laboratory findings						
LV ejection fraction, %	56.1 ± 9.1	57.7 ± 8.0	56.0 ± 9.1	54.8 ± 9.5	53.2 ± 10.6	<0.001
WBC, x 10^3^/mm^3^	8.9 ± 3.5	8.4 ± 3.4	8.9 ± 3.6	9.3 ± 3.5	9.8 ± 3.7	<0.001
Hemoglobin, g/dL	13.4 ± 1.9	13.7 ± 1.8	13.5 ± 1.8	13.3 ± 2.0	13.0 ± 2.2	<0.001
Platelet, x 10^3^/mm^3^	238.5 ± 69.4	234.4 ± 63.1	237.7 ± 63.6	240.3 ± 73.8	248.2 ± 82.4	<0.001
GFR (MDRD), mL/min/1.73 m^2^	86.2 ± 29.8	89.6 ± 26.5	85.6 ± 28.7	86.2 ± 31.0	77.3 ± 35.2	<0.001
Total cholesterol, mg/dL	179.2 ± 47.8	176.8 ± 45.9	181.8 ± 47.0	178.7 ± 48.4	184.8 ± 52.0	<0.001
LDL cholesterol, mg/dL	115.9 ± 42.4	113.6 ± 41.0	119.8 ± 43.6	115.9 ± 43.3	120.2 ± 43.9	<0.001
HDL cholesterol, mg/dL	44.9 ± 13.7	45.7 ± 13.3	45.0 ± 12.0	44.5 ± 13.7	43.0 ± 15.4	<0.001
HbA_1c_, %	6.4 ± 1.3	6.4 ± 1.3	6.4 ± 1.2	6.5 ± 1.4	6.6 ± 1.4	<0.001
Procedural characteristics						
AHA/ACC lesion: type B2/C	4,026 (88.3)	2022 (87.0)	415 (88.7)	841 (90.0)	748 (89.6)	<0.001
Multivessel disease	2,128 (46.6)	1005 (43.2)	217 (46.4)	455 (48.7)	451 (54.0)	<0.001
Multivessel PCI	861 (18.9)	393 (16.9)	93 (19.9)	198 (21.2)	177 (21.2)	0.006
Target lesion						0.634
Left main coronary artery	43 (0.9)	20 (0.9)	3 (0.6)	12 (1.3)	8 (1.0)	
Left anterior descending artery	2,174 (47.7)	1128 (48.5)	210 (44.9)	447 (47.9)	389 (46.6)	
Left circumflex artery	905 (19.8)	455 (19.6)	111 (23.7)	184 (19.7)	155 (18.6)	
Right coronary artery	1,435 (31.5)	719 (30.9)	144 (30.8)	290 (31.0)	282 (33.8)	
Others	5 (0.1)	3 (0.1)	0 (0.0)	1 (0.1)	1 (0.1)	
Treatment method						0.405
Drug-eluting stent	4,085 (89.5)	2071 (89.1)	423 (90.4)	839 (89.8)	752 (90.1)	
Bioresorbable scaffold	79 (1.7)	48 (2.1)	8 (1.7)	15 (1.6)	8 (1.0)	
Bare metal stent	18 (0.4)	13 (0.6)	0 (0.0)	2 (0.2)	3 (0.4)	
Drug-coated balloon	148 (3.2)	83 (3.6)	12 (2.6)	30 (3.2)	23 (2.8)	
POBA	232 (5.1)	110 (4.7)	25 (5.3)	48 (5.1)	49 (5.9)	
Number of stents	1.5 ± 0.8	1.4 ± 0.7	1.5 ± 0.8	1.5 ± 0.8	1.5 ± 0.8	<0.001
Stent length, mm	37.5 ± 22.7	35.8 ± 21.9	36.9 ± 22.0	39.7 ± 23.4	40.1 ± 24.1	<0.001
Stent diameter, mm	3.2 ± 0.5	3.2 ± 0.5	3.2 ± 0.5	3.1 ± 0.5	3.1 ± 0.5	0.014
Concomitant medications						
Aspirin 100 mg qd	4,504 (98.7)	2,299 (98.9)	457 (97.6)	925 (99.0)	823 (98.6)	0.129
Type of P2Y_12_ inhibitor						0.950
Clopidogrel 75 mg qd	3,654 (80.1)	1,861 (80.0)	369 (78.8)	750 (80.3)	674 (80.7)	
Prasugrel	208 (4.6)	101 (4.3)	27 (5.8)	42 (4.5)	38 (4.6)	
Ticagrelor	614 (13.5)	322 (13.8)	64 (13.7)	122 (13.1)	106 (12.7)	
Beta blocker	2,961 (64.9)	1,479 (63.6)	273 (58.3)	640 (68.5)	569 (68.1)	<0.001
Angiotensin blockade	3,402 (74.6)	1,732 (74.5)	323 (69.0)	717 (76.8)	630 (75.4)	0.016
Calcium channel blocker	328 (7.2)	186 (8.0)	33 (7.1)	56 (6.0)	53 (6.3)	0.159
Statin	4,335 (95.0)	2,223 (95.6)	439 (93.8)	893 (95.6)	780 (93.4)	0.038
Proton pump inhibitor	2,744 (60.1)	1,333 (57.3)	302 (64.5)	572 (61.2)	537 (64.3)	<0.001

Values are mean ± SD, median (IQR), or n (%).

ACC = American College of Cardiology; AHA = American Heart Association; CABG = coronary artery bypass graft; CKD = chronic kidney disease; GFR = glomerular filtration rate; HbA_1c_ = hemoglobin A1c; HDL = high-density lipoprotein; hsCRP = high-sensitivity C-reactive protein; LDL = low density lipoprotein; LV = left ventricular; NSTEMI = non–ST-segment elevation myocardial infarction; PCI = percutaneous coronary intervention; POBA = plain optimal balloon angioplasty; RIR = residual inflammatory risk; STEMI = ST-segment elevation myocardial infarction; WBC = white blood count.

**Table 2:** The Determinants of Persistent High RIR vs Other RIRs

	Univariable Analysis		Multivariable Analysis	
	OR (95% CI)	*P* Value	OR (95% CI)	*P* value
Index presentation with MI	1.44 (1.23-1.68)	<0.001	-	-
Age (per 1-y increase)	1.02 (1.01-1.02)	<0.001	1.02 (1.01-1.02)	<0.001
Smoking	1.35 (1.16-1.58)	<0.001	1.57 (1.30-1.89)	<0.001
Diabetes mellitus	1.19 (1.00-1.40)	0.032	-	-
Chronic kidney disease	2.55 (2.13-3.04)	<0.001	1.91 (1.55-2.35)	<0.001
Hemoglobin (per 1 g/dL increase)	0.86 (0.83-0.90)	<0.001	0.90 (0.85-0.94)	<0.001
Previous stroke	1.35 (1.02-1.76)	0.033	-	-
WBC (per 10^3^/mm^3^ increase)	1.08 (1.06-1.11)	<0.001	1.07 (1.04-1.09)	<0.001
LDL cholesterol (per 1 mg/dL increase)	1.00 (1.00-1.01)	0.002	1.00 (1.00-1.01)	<0.001
HDL cholesterol (per 1 mg/dL increase)	0.99 (0.98-0.99)	<0.001	0.99 (0.98-0.99)	<0.001
Multivessel disease	1.44 (1.24-1.67)	<0.001	1.30 (1.10-1.52)	0.002
Medication: statin	0.69 (0.51-0.95)	0.019	0.65 (0.46-0.92)	0.016

MI = myocardial infarction; other abbreviations as in Table 1.

### Clinical outcomes according to type of residual inflammatory risk

**Figure 3:**
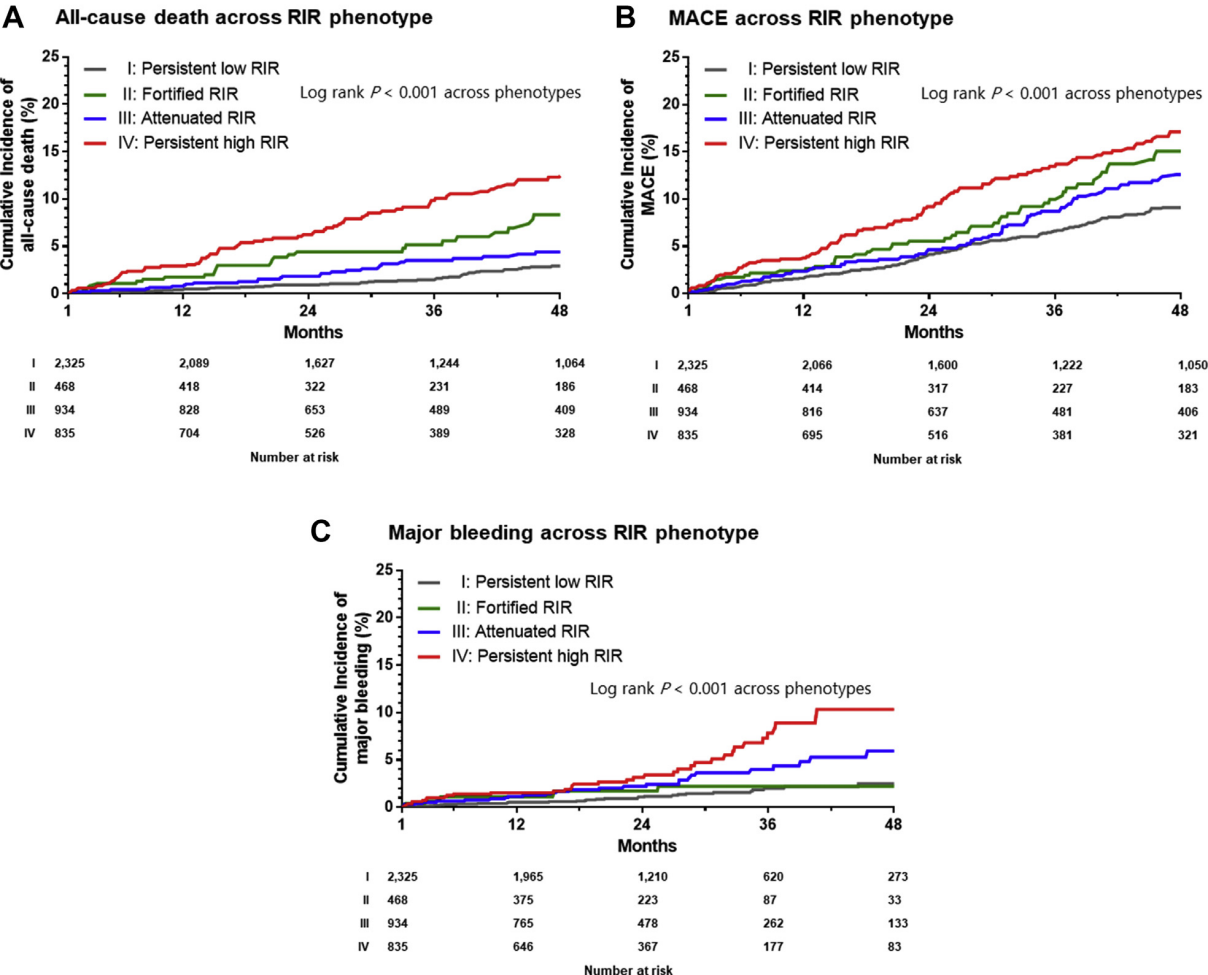
Kaplan-Meier Curves for Adverse Clinical Events, Stratified by Phenotype of Residual Inflammatory Risk Abbreviations as in Figure 1.

During the follow-up period, there were significant differences in the risks of all-cause death, MACE, and major bleeding across the RIR groups (all *P* < 0.001) (Table 3, Figure 3). When adjusted with known important covariates, patients with persistent high RIR showed significantly increased rates of all-cause death (HR: 2.16; 95% CI: 1.54 to 3.03; *P* < 0.001), MACE (HR: 1.41; 95% CI: 1.12 to 1.78; *P* = 0.004), and major bleeding (HR: 2.58; 95% CI: 1.57 to 4.23; *P* < 0.001) compared with those with persistent low RIR (Table 4).

**Table 3:** Clinical Outcomes According to Phenotype of Residual Inflammatory Risk

	Overall Population (N = 4,562)	Persistent Low RIR (n = 2,325)	Fortified RIR (n = 468)	Attenuated RIR (n = 934)	Persistent High RIR (n = 835)	*P* Value
All-cause death	238 (5.2)	71 (3.1)	34 (7.3)	47 (5.0)	86 (10.3)	<0.001
MACE	522 (11.4)	217 (9.3)	66 (14.1)	115 (12.3)	124 (14.9)	<0.001
CV death	92 (2.0)	28 (1.2)	15 (3.2)	17 (1.8)	32 (3.8)	<0.001
Nonfatal MI	272 (6.0)	122 (5.2)	27 (5.8)	71 (7.6)	52 (6.2)	0.081
Nonfatal stroke	158 (3.5)	67 (2.9)	24 (5.1)	27 (2.9)	40 (4.8)	0.009
Major bleeding	111 (2.4)	32 (1.4)	9 (1.9)	31 (3.3)	39 (4.7)	<0.001

Values are n (%). Major adverse cardiac events (MACE) included CV death, nonfatal MI, and nonfatal stroke.

Abbreviations as in Table 1.

**Table 4:** Hazard Ratio for Clinical Outcomes According to Risk Groups

Phenotype of Residual Inflammatory Risk (Reference: Persistent Low RIR)							
Events	Group	Unadjusted Model			Adjusted Model^a^		
		HR	95% CI	*P* Value	HR	95% CI	*P* Value
All-cause death	Fortified RIR	2.60	1.73-3.92	<0.001	1.64	1.07-2.53	0.024
	Attenuated RIR	1.68	1.16-2.43	0.006	1.20	0.83-1.75	0.336
	Persistent high RIR	3.80	2.77-5.20	<0.001	2.16	1.54-3.03	<0.001
MACE	Fortified RIR	1.68	1.27-2.21	<0.001	1.49	1.12-1.98	0.006
	Attenuated RIR	1.36	1.08-1.70	0.008	1.21	0.96-1.52	0.109
	Persistent high RIR	1.81	1.45-2.25	<0.001	1.41	1.12-1.78	0.004
Major bleeding	Fortified RIR	1.61	1.61-3.38	<0.001	1.26	0.60-2.67	0.549
	Attenuated RIR	2.37	1.44-3.88	0.006	1.85	1.11-3.08	0.018
	Persistent high RIR	3.87	2.42-6.18	<0.001	2.58	1.57-4.23	<0.001

Persistent High RIR vs Other RIRs								
Events	Rates		Unadjusted Model			Adjusted Model^a^		
	Persistent High RIR (n = 835)	Other RIRs (n = 3,727)	HR	95% CI	*P* Value	HR	95% CI	*P* Value
All-cause death	86 (10.3)	152 (4.1)	2.80	2.15-3.65	<0.001	1.92	1.44-2.55	<0.001
MACE	124 (14.9)	398 (10.7)	1.55	1.27-1.89	<0.001	1.26	1.02-1.56	0.032
CV death	32 (3.8)	60 (1.6)	2.63	1.71-4.04	<0.001	1.59	1.00-2.53	0.051
Non-fatal MI	52 (6.2)	220 (5.9)	1.16	0.81-1.66	0.421	1.00	0.70-1.45	0.983
Non-fatal stroke	40 (4.8)	72 (1.6)	1.56	1.14-2.15	0.006	1.37	0.98-1.92	0.066
Major bleeding	39 (4.7)	58 (1.6)	2.72	1.84-4.02	<0.001	1.98	1.30-2.99	<0.001

Values are n (%). Major adverse cardiac events (MACE) included CV death, nonfatal MI, and nonfatal stroke.

a Adjusted for index myocardial infarction (MI) presentation, age, gender, body mass index, smoking, diabetes, hypertension, dyslipidemia, chronic kidney disease, anemia, previous stroke, left ventricular ejection fraction, percutaneous coronary intervention for left anterior descending artery lesion, multivessel disease, potent P2Y_12_ inhibitor, beta-blocker, angiotensin blockade, and statin.

**Figure 4:**
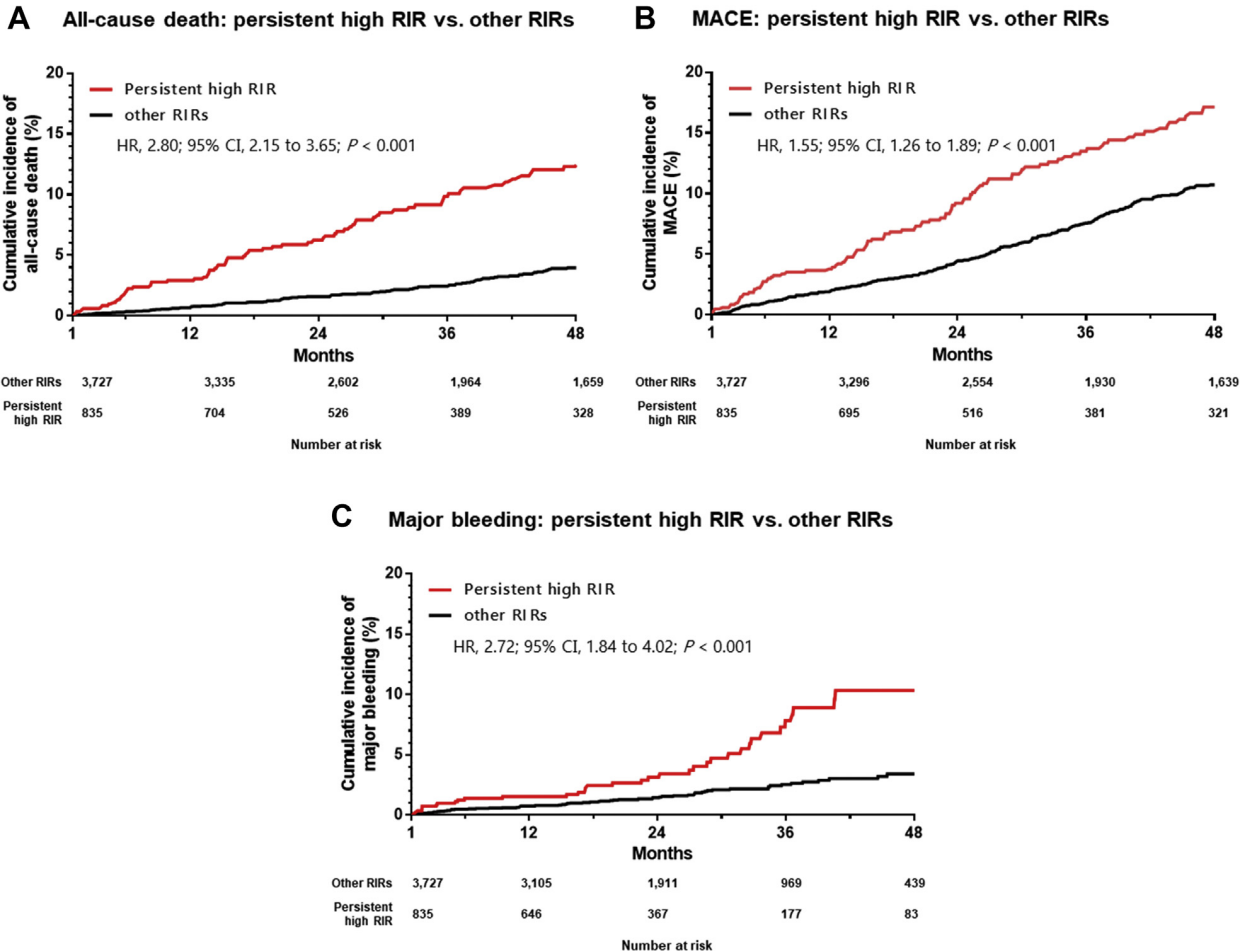
Kaplan-Meier Curves for Adverse Clinical Events Between Persistent High RIR vs Other RIRs Abbreviations as in Figure 1.

For the next step, we evaluated the prognostic implication of persistent high RIR in this cohort. Persistent high RIR was significantly associated with higher incidence of all-cause death (adjusted HR: 1.92; 95% CI: 1.44 to 2.55; *P* < 0.001), MACE (adjusted HR: 1.26; 95% CI: 1.02 to 1.56; *P* = 0.032), and major bleeding (adjusted HR: 1.98; 95% CI: 1.30 to 2.99; *P* < 0.001), the findings of which remained consistent in the fully adjusted model (Figure 4, Table 4).

### Impact of persistent high RIR according to subgroups

**Figure 5:**
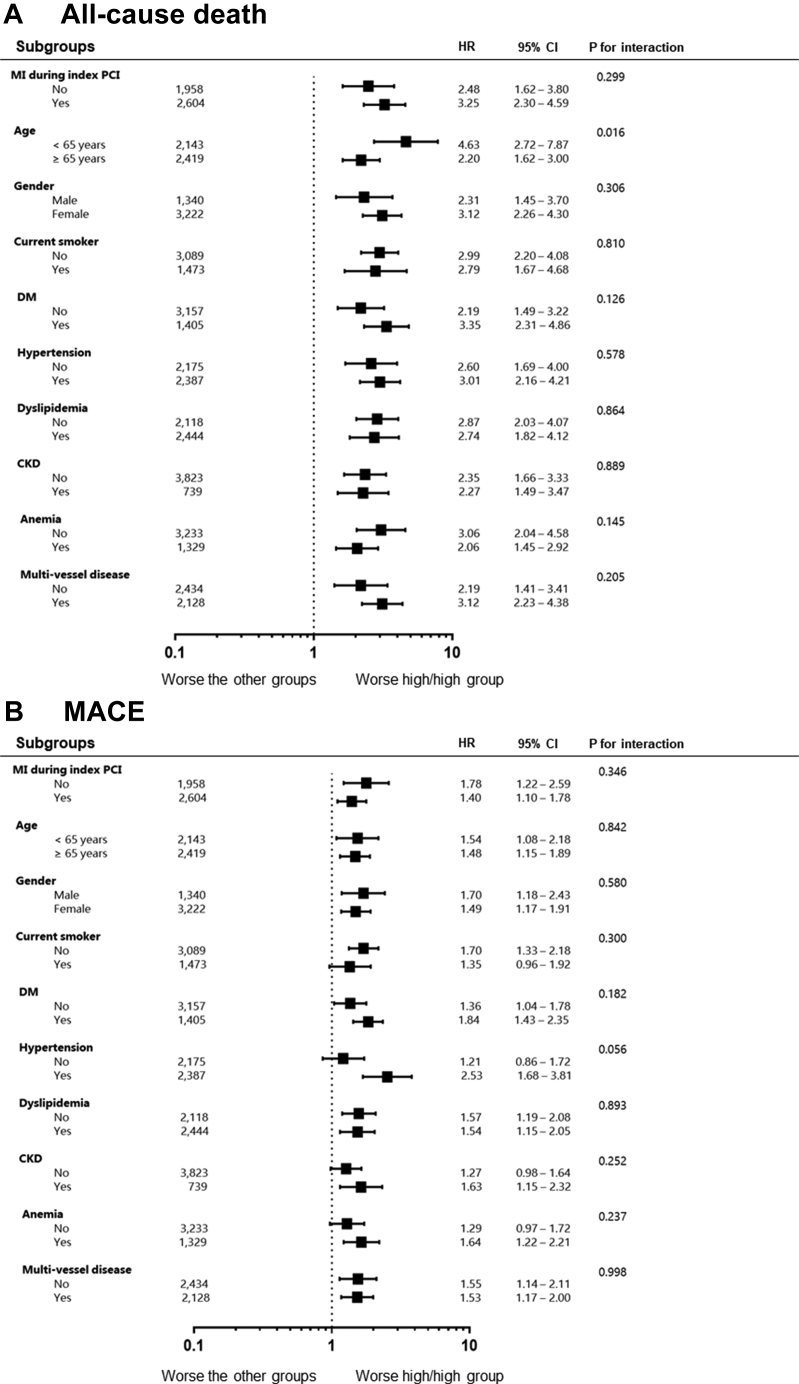

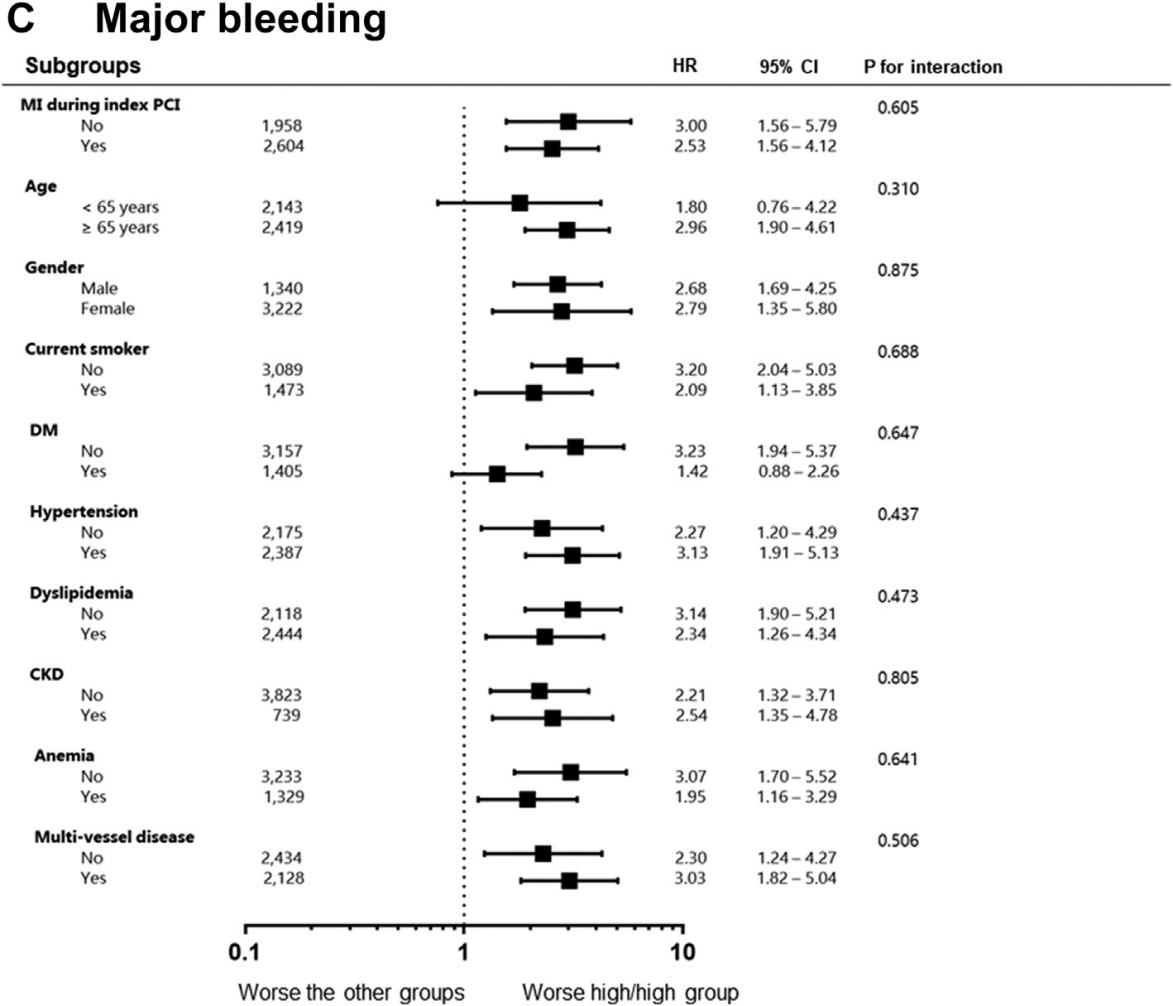
Comparative Hazard Ratios of Adverse Clinical Events Across Subgroups CKD = chronic kidney disease; DM = diabetes mellitus; other abbreviations as in Figures 1 and 2.

We evaluated the clinical impact of persistent high RIR on the occurrences of all-cause death, MACE, and major bleeding across the subgroups (Figure 5). Compared with patients without persistent high RIR, subjects with persistent high RIR showed the worse outcomes in all-cause death, MACE, and major bleeding across all subgroups. The significant interaction was observed only in the relationships between age and all-cause death (*P* = 0.016), in which the magnitude was significantly larger among younger patients.

## Discussion

This analysis is the first analysis to evaluate the prognostic implication of early-determined RIR phenotype (on admission and at 1 month post-PCI) in East Asian patients with significant CAD. The principal findings of this study are as follows: 1) prevalence of persistent high RIR (hsCRP ≥2 mg/L on admission and at 1 month: 18.3% of the total cohort) was relatively low in this East Asian registry; 2) persistent high RIR was significantly associated with baseline characteristics (age, smoking, CKD, hemoglobin, WBC count, cholesterol level, multivessel disease) and a concomitant medication (statin); and 3) early determined phenotype of persistent high RIR showed the consistent associations with adverse CV outcomes including ischemic and bleeding events. Previous reports assessed inflammation status based on serial hsCRP measurements with long-time interval (about 35 to 56 weeks),^14^^,^^16^ whereas the current PCI registry suggested the prognostic implication in early determination of inflammation status (approximately 4 weeks). The latter strategy can give PCI-treated patients more clinical benefits, as it may determine the appropriate patients with RIR earlier and guide the introduction of anti-inflammatory therapy faster.

### Association between inflammation status and prognosis in patients with CAD

Among patients with stable CAD^23^^,^^24^ or ACS,^25^, ^26^, ^27^ a strong correlation between hsCRP measured at baseline and future CV events has been demonstrated in most studies. In patients with ACS, the elevated phenotype of hsCRP measurement can not only be a marker of widespread underlying vascular inflammation but also be a strong predictor for a worse short- and long-term clinical prognosis.

In patients with ACS achieving recommended LDL-cholesterol levels with intensive statin treatment, quite a few subjects still experience ischemic events. One important target to reduce this residual CV risk has focused on addressing inflammation. In post hoc analyses of the PROVE IT-TIMI 22 (Pravastatin or Atorvastatin Evaluation and Infection Therapy-Thrombolysis In Myocardial Infarction 22) trial including stabilized patients with ACS (n = 3,745),^28^ the proportion of RIR (on statin hsCRP ≥2 mg/L) was 43%. Even after statin treatment, the risk of coronary death and recurrent MI was increased in those with hsCRP ≥2 mg/L vs hsCRP <2 mg/L (3.9 vs 2.8 events per 100 person-years, *P* = 0.006). Similar data were observed in the IMPROVE-IT (Improved Reduction of Outcomes: Vytorin Efficacy International) trial including 15,179 patients stabilized after ACS.^29^ At 1 month after randomization, 39% achieved the dual LDL-cholesterol (<70 mg/dL) and hsCRP (<2 mg/L) targets, 14% met neither target, 14% met only the hsCRP target, and 33% met only the LDL-cholesterol target. Achievement of hsCRP target (<2 mg/L) only was associated with an 11% lower relative risk in the primary endpoint in comparison with meeting neither target (adjusted HR: 0.89; 95% CI: 0.79 to 0.99; *P* = 0.041).

### Emerging role of anti-inflammatory therapy in cardiovascular disease

Several clinical trials have suggested direct evidence of pharmacologic anti-inflammatory intervention to improve clinical outcomes in patients with ASCVD.^30^ These clinical trials have transformed influence of inflammation on atherosclerotic progression from theory to practice. The CANTOS (Canakinumab Anti-inflammatory Thrombosis Outcomes Study) demonstrated that interleukin (IL)-1ß antagonist, canakinumab at a dose of 150 mg every 3 months, reduced composite ischemic events (HR: 0.85; 95% CI: 0.74 to 0.98; *P* = 0.021) in patients at least 1 month post-MI with hsCRP ≥2 mg/L.^10^ Two large-scale clinical studies have shown that colchicine treatment—a microtubule inhibitor that putatively decreases the level of hsCRP and IL-6—can reduce recurrent CV events. The Colchicine Cardiovascular Outcomes Trial (COLCOT), including 4,745 patients within a median of 14 days following AMI, demonstrated 33% reduction in occurrence of ischemic events (5.5% in the colchicine group vs 7.1% in the placebo group: HR: 0.77; 95% CI: 0.61 to 0.96; *P* = 0.02).^11^ Likewise, the benefit of low-dose colchicine (0.5 mg once daily) has been proven among patients with chronic coronary syndrome (2.5 vs 3.6 ischemic events per 100 person-years in the colchicine vs placebo group; HR: 0.69; 95% CI: 0.57 to 0.83; *P* < 0.001).^12^ In contrast, the CIRT (Cardiovascular Inflammation Reduction Trial) failed to demonstrate clinical benefit of low-dose methotrexate in secondary prevention. The level of hsCRP measurement was very low (median, 1.5 mg/L), and methotrexate overall had limited effect on controlling IL-1ß, IL-6, or hsCRP.^31^

Much work remains to optimize further anti-inflammatory interventions, minimize unwanted actions, and refine patient selection.^30^ In this context, reliable biomarkers (eg, hsCRP) may give great hope to point the right patient, with the right therapy, at the right time: the tenets of precision medicine.^13^ This biomarker-based approach and opens a new avenue to reducing CV risk that remains despite current guideline-recommended treatments for ASCVD.

### Temporal variability of inflammation status and importance of serial assessment

Previous clinical registries mostly decided the inflammatory risk based on hsCRP at admission under the limited effects of concomitant medications (eg, statins).^32^^,^^33^ Otherwise, post hoc analysis from randomized clinical trials for statin strategies determined the risk of RIR at approximately 1 to 3 months on statins.^1^^,^^13^^,^^28^^,^^30^ The current analysis demonstrated that hsCRP criteria at 1 month on treatment vs on admission showed more statistical power for predicting ischemic events in PCI-treated patients.

Contrary to LDL-cholesterol with on-treatment stable value, inflammatory level appears variable over time. The latter finding may be related with its tendency to increase easily with various stimuli, between-patient variability in the balance of underlying mechanisms contributing to atheroprogression, and various responses to current medications with anti-inflammatory effects.^13^ Local and systemic inflammation may precipitate atherothrombosis as well as increase according to the extent of myocardial damage following MI. In patients presented with AMI, hsCRP rises rapidly, peaking at 2 to 4 days.^25^ After several weeks, hsCRP gradually returns to baseline but remains elevated in some patients for longer periods.^13^ To use hsCRP as a reliable RIR biomarker, its measurement may be delayed for at least 4 to 6 weeks after an MI to permit resolution of the acute-phase reaction.^34^

The recent PCI data including our registry data have suggested clinical usefulness of RIR assessment based on serial hsCRP measurement,^14^^,^^16^ which may reduce the risk of misunderstanding for the realistic RIR phenotype. Compared with other RIR phenotypes, persistent high RIR (hsCRP ≥2 mg/L both on admission and at follow-up) showed the strongest association with occurrence of ischemic events following PCI. Previous analysis data have some issues for clinical application,^14^^,^^16^ as their time interval between hsCRP measurements was long and nonconstant (56.0 ± 78.2 weeks in the American cohort and 34.7 ± 3.7 weeks in the Japanese cohort). We classified RIR phenotype based on hsCRP measurements with a relatively constant time interval: a strategy that can maximize clinical benefit by introduction of anti-inflammatory therapy during the early phase with a higher risk of ischemic events.

### Inflammation: a hidden key to explain racial differences in clinical prognoses

An increasing body of evidence demonstrates that East Asian patients have a lower risk of atherothrombotic events and a higher tendency of serious bleeding during antithrombotic treatment compared with Caucasian patients.^18^ In a recent meta-analysis including PCI-treated patients (n = 16,518), ischemic events occurred more frequently in non-East Asians (0.8% vs 1.8%; *P* < 0.001), whereas major bleeding events occurred more frequently in East Asians (0.6% vs 0.3%, *P* = 0.001).^17^ East Asians show a lower level of intrinsic thrombogenicity (eg, inflammation and coagulation activity) compared with Caucasians, which may, in part, explain the lower morbidity and mortality associated with ASCVD in East Asians compared with Westerners.^19^

There are significant racial differences in inflammatory activity. Overall, African Americans exhibit the highest, and East Asians appear to have the lowest levels of inflammation.^19^ A recent large pooled database of 10 randomized clinical trials (n = 22,638) assessed the race-based difference of ischemic endpoint in PCI-treated patients. Five-year major CV event rates were 18.8% in White patients (reference group), compared with 23.9% in Black patients (*P* = 0.0009), 11.2% in Asian patients (*P* = 0.0007), and 21.5% in Hispanic patients (*P* = 0.07). Multivariate analysis demonstrated an independent association between black race and occurrence of CV events (HR: 1.28; 95% CI: 1.05 to 1.57; *P* = 0.01).^35^

After guideline-recommended treatment, Western clinical data showed a higher rate of enhanced RIR (on-treatment hsCRP ≥2 mg/L) up to 43% to 61% in patients with CAD.^10^^,^^28^^,^^29^ In the American PCI cohort,^14^ median values of hsCRP were 2.2 (IQR: 0.9 to 5.4) mg/L at admission and 1.8 (IQR: 0.8 to 4.4) mg/L at the last follow-up, and persistent high RIR was observed in 36.5% (Central Illustration). In our Korean registry, their median values were 1.2 (IQR: 0.5 to 3.7) mg/L on admission and 0.9 (IQR: 0.5 to 2.3) mg/L at 1 month, and persistent high RIR was observed in 18.3% (∼50% compared with the American registry). This difference would be another important piece of biological evidence to support better clinical outcomes in East Asian patients with significant CAD.

**Central Illustration:**
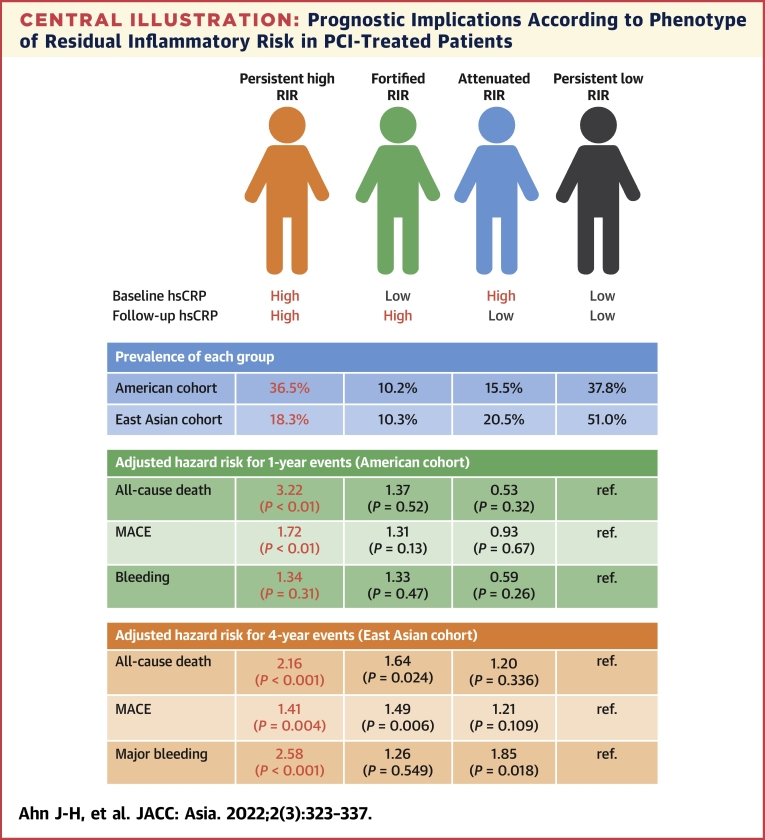
Prognostic Implications According to Phenotype of Residual Inflammatory Risk in PCI-Treated Patients Residual inflammatory risk (RIR) was determined by serial measurements of high-sensitivity C-reactive protein (hsCRP) at on-admission and 1-month follow-up. Compared with American cohort (Mount Sinai Hospital registry), East Asian cohort (GNUH registry) had a lower prevalence of persistent high RIR (18.3% vs 36.5%). Compared with other RIR phenotypes, persistent high RIR phenotype showed higher risks of all-cause death and major bleeding in patients undergoing percutaneous coronary intervention (PCI). MACE = major adverse cardiovascular events.

### Study limitations

First, this is a retrospective analysis from a prospective registry, but hsCRP measurements were performed with relatively consistent time intervals. Second, considerable portion of patients (approximately 20%) were excluded from this analysis because there were unmeasured 1-month hsCRPs. Third, we used the criteria of hsCRP (≥2 mg/L) based on the previous report from the Western registry;^14^ therefore, these criteria can be different with the realistic high-risk cutoff in East Asian patients. Finally, hsCRP levels may be affected by certain clinical situations such as infection and other inflammatory disease. Information regarding these entities was not available in the current study.

## Conclusions

This is the first study to show that early determination of RIR status can help to choose the appropriate high-risk patients undergoing PCI. Approximately one-fifth of East Asian patients with CAD have persistent high RIR, which appears to have distinct impact on ischemic and bleeding events. The unique RIR profiles of these patients may partly explain their better clinical outcomes compared with Western populations.

Perspectives **COMPETENCY IN PATIENT CARE:** Clinical evidences have supported usefulness of measuring hsCRP in assessing level of inflammation and predicting clinical outcomes in healthy individuals and patients with cardiovascular disease. Because inflammatory levels appear variable over time, serial hsCRP assessment has been suggested to determine the reliable residual inflammatory risk phenotype in high-risk patients. In the American cohort including patients treated with percutaneous coronary intervention, persistent high RIR (hsCRP ≥2 mg/L by serial measurements) was observed in ∼40%, the phenotype of which was significantly associated with the risks of all-cause death and ischemic events.

**TRANSLATIONAL OUTLOOK:** In the East Asian PCI registry, prevalence of persistent high RIR (hsCRP ≥2 mg/L on admission and at 1 month) was relatively low (∼18.3% of the total cohort). Persistent high RIR was significantly associated with multiple covariates (eg, CV risk factors, laboratory measurements, and concomitant medication). Early determination of persistent high RIR was significantly associated with the prevalence of major bleeding as well as the risks of all-cause death and ischemic events.

## Funding Support and Author Disclosures

This study was supported by research grants from the Basic Science Research Program through the National Research Foundation (NRF) of Korea, funded by the Ministry of Science, ICT, and Future Planning (NRF-2015R1A5A2008833). Dr Gurbel has received grants and personal fees from Bayer HealthCare LLC, Amgen, Janssen, U.S. WorldMeds LLC, and Otitopic Inc; grants from Instrumentation Laboratory, Hikari Dx, Haemonetics, Medicure Inc., and Idorsia Pharmaceuticals; personal fees from Up-To-Date outside the submitted work; in addition, Dr Gurbel holds patents for Detection of Restenosis Risk in Patients and and Assessment of Cardiac Health and Thrombotic Risk in a Patient. Dr Jeong has received honoraria for lectures from AstraZeneca, Daiichi Sankyo, Sanofi-Aventis, Han-mi Pharmaceuticals, and Yuhan Pharmaceuticals and research grants or support from Yuhan Pharmaceuticals and U&I Corporation. All other authors have reported that they have no relationships relevant to the contents of this paper.
